# A Genome-Wide CRISPR/Cas9-Based Screen Identifies Heparan Sulfate Proteoglycans as Ligands of Killer-Cell Immunoglobulin-Like Receptors

**DOI:** 10.3389/fimmu.2021.798235

**Published:** 2021-11-30

**Authors:** Klara Klein, Angelique Hölzemer, Tim Wang, Tae-Eun Kim, Haley L. Dugan, Stephanie Jost, Marcus Altfeld, Wilfredo F. Garcia-Beltran

**Affiliations:** ^1^ Broad Institute of MIT and Harvard, Cambridge, MA, United States; ^2^ Whitehead Institute for Biomedical Research, Cambridge, MA, United States; ^3^ Institute of Pharmacology and Toxicology, University of Veterinary Medicine Vienna, Vienna, Austria; ^4^ Leibniz Institute for Experimental Virology, Hamburg, Germany; ^5^ First Department of Internal Medicine, Division of Infectious Diseases, University Medical Centre Eppendorf, Hamburg, Germany; ^6^ German Center for Infection Research (DZIF), Site Hamburg-Lübeck-Borstel-Riems, Hamburg, Germany; ^7^ Department of Biology, Massachusetts Institute of Technology, Cambridge, MA, United States; ^8^ Ragon Institute of Massachusetts General Hospital (MGH), MIT, and Harvard, Cambridge, MA, United States; ^9^ Adimab, LLC, Lebanon, NH, United States; ^10^ Center for Virology and Vaccine Research, Beth Israel Deaconess Medical Center, Boston, MA, United States; ^11^ Department of Pathology, Massachusetts General Hospital (MGH), Boston, MA, United States

**Keywords:** CRISPR, screen, KIR, heparan sulfate, NK cells

## Abstract

While human leukocyte antigen (HLA) and HLA-like proteins comprise an overwhelming majority of known ligands for NK-cell receptors, the interactions of NK-cell receptors with non-conventional ligands, particularly carbohydrate antigens, is less well described. We previously found through a bead-based HLA screen that KIR3DS1, a formerly orphan member of the killer-cell immunoglobulin-like receptor (KIR) family, binds to HLA-F. In this study, we assessed the ligand binding profile of KIR3DS1 to cell lines using Fc fusion constructs, and discovered that KIR3DS1-Fc exhibited binding to several human cell lines including ones devoid of HLA. To identify these non-HLA ligands, we developed a magnetic enrichment-based genome-wide CRISPR/Cas9 knock-out screen approach, and identified enzymes involved in the biosynthesis of heparan sulfate as crucial for the binding of KIR3DS1-Fc to K562 cells. This interaction between KIR3DS1 and heparan sulfate was confirmed *via* surface plasmon resonance, and removal of heparan sulfate proteoglycans from cell surfaces abolished KIR3DS1-Fc binding. Testing of additional KIR-Fc constructs demonstrated that KIR family members containing a D0 domain (KIR3DS1, KIR3DL1, KIR3DL2, KIR2DL4, and KIR2DL5) bound to heparan sulfate, while those without a D0 domain (KIR2DL1, KIR2DL2, KIR2DL3, and KIR2DS4) did not. Overall, this study demonstrates the use of a genome-wide CRISPR/Cas9 knock-out strategy to unbiasedly identify unconventional ligands of NK-cell receptors. Furthermore, we uncover a previously underrecognized binding of various activating and inhibitory KIRs to heparan sulfate proteoglycans that may play a role in NK-cell receptor signaling and target-cell recognition.

## Introduction

NK-cell function is dictated by germline-encoded activating and inhibitory receptors that interact with a plethora of ligands expressed on target cells (1). Major classes of NK-cell receptors are (1) natural cytotoxicity receptors (NCRs), which are comprised of NKp30, NKp44, and NKp46, (2) killer-cell lectin-like receptors (KLRs), which include NKG2A, NKG2C, and NKG2D, and (3) killer-cell immunoglobulin-like receptors (KIRs), the most polymorphic family of NK-cell receptors (2). These receptors allow NK cells to discriminate between healthy “self” and a variety of pathological cell states (3). In light of the associations of several NK-cell receptors with protective or detrimental effects in human diseases, current research efforts continue to uncover novel ligands for NK-cell receptors and further characterize the specificity of known interactions. However, an overwhelming number of these ligands arise from known families of proteins, such as human leukocyte antigen (HLA) and other HLA-like proteins, leaving a major gap in our knowledge of the role of unconventional ligands for NK-cell receptors.

An important group of unconventional ligands are carbohydrates expressed on target cells. Indeed, all cells in the body bear a glycocalyx, a term used to refer to the entire array of glycans coating the cell surface (4). A major component of the glycocalyx is glycosaminoglycans (GAGs), which are long, unbranched polysaccharides that can be extensively modified by acetyl and sulfate substitutions in an intricate, diverse, and tightly regulated manner on individual cells (5). One of the most abundant GAGs is heparan sulfate, which is made by every cell in the body and plays a multitude of biological roles [reviewed in (6)] including wound repair (7, 8), tumorigenesis (9), cell adhesion and migration (10), signaling of growth factors (11, 12) and cytokines (13, 14), and host defense (15). The carbohydrate backbone of heparan sulfate is synthesized and covalently attached to protein cores by several enzymes (i.e. XYLT1/2, B4GALT7, B4GALT6, B3GAT3, EXT1/2, EXTL1/2/3) and subsequently modified by epimerases (i.e. GLCE), sulfotransferases (i.e. NDST1/2/3/4, HS6ST1/2/3, HS2ST1, and HS3ST1/2/3/4/5/6), and sulfatases (i.e. SULF1/2). While the most well-known form of heparan sulfate is heparin—a soluble, highly sulfated form of heparan sulfate with anti-coagulant properties—by far the most prevalent form of heparan sulfate is found as a proteoglycan on cell surfaces. Much like DNA or RNA, heparan sulfate does not have a single structure or sequence, but rather, the polysaccharide components that make heparan sulfate vary depending on the cell type and cellular context under which they are made. Thus, heparan sulfate has been referred to as “the most information-dense biopolymers found in nature” (16).

Among its many biological functions, heparan sulfate has been shown to interact with numerous soluble and membrane-bound proteins, including certain NK-cell receptors. NCRs, which are widely expressed on NK cells (17), have been shown to bind to specific heparan sulfate motifs. NKp30 and NKp46 bind to epitopes containing 2-*O*-sulfated iduronic acid and 6-*O*- and *N*-sulfated glucosamine, while NKp44 binds to epitopes containing 2-*O*-sulfated iduronic acid and *N*-acetylated glucosamine (18). On the other hand, KIR2DL4, a mixed activating and inhibitory KIR, was shown to bind heparan sulfate in a manner that depended on 3-*O*-sulfation and that directly influenced KIR2DL4 signaling and NK-cell function (19). Assessment of other KIRs has not yet been performed.

In this study, we used a magnetic enrichment-based genome-wide CRISPR/Cas9 knock-out screen to investigate non-HLA ligands for KIRs. Using a soluble construct of KIR3DS1, an activating NK-cell receptor previously shown by our group to bind HLA-F (20), we found that enzymes involved in the biosynthesis of heparan sulfate were critical for generating KIR3DS1 ligands on the cell surface of HLA-deficient cells (K562 cells). Binding of KIR3DS1 to heparan sulfate was confirmed *via* surface plasmon resonance and could be abolished by eliminating heparan sulfate proteoglycans in several cell lines. Further testing of additional KIRs demonstrated that KIRs bearing a D0 domain also bound to heparan sulfate. These findings uncover heparan sulfate proteoglycans as novel ligands for KIRs, which may play influential roles in NK-cell receptor signaling and recognition of target cells.

## Results

### KIR3DS1 Ligands Are Expressed in Many Tumor Cell Lines, Including Those Devoid of HLA

**Figure 1:**
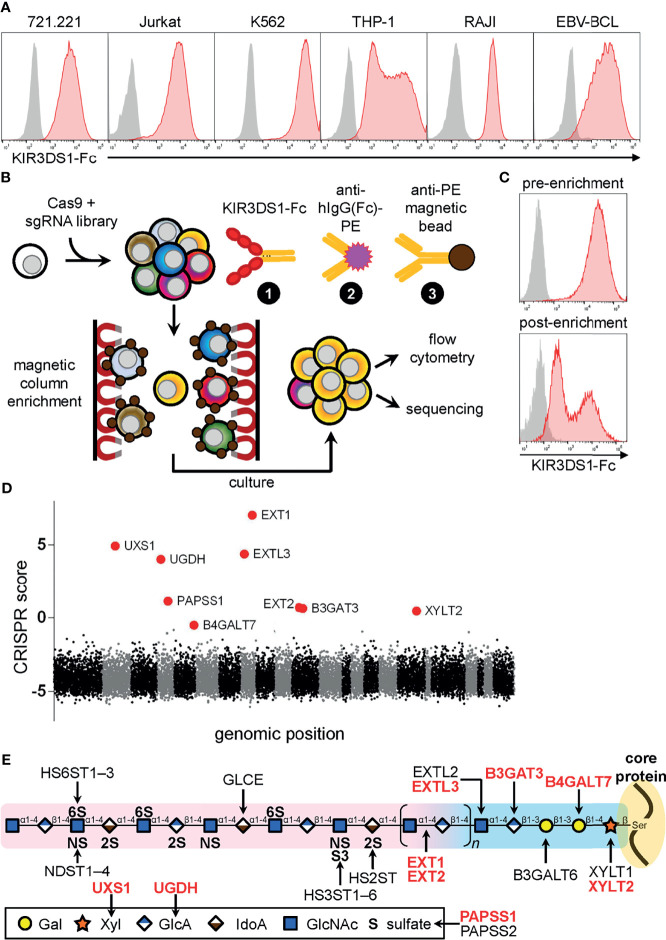
Genome-wide CRISPR/Cas9-based screen for KIR3DS1 ligands identifies heparan sulfate biosynthesis pathway genes. **(A)** The indicated human cell lines were stained with KIR3DS1-Fc and anti-human IgG Fc secondary antibody (red histograms) or only with secondary antibody as a control (gray histograms). **(B)** Schematic illustration of the genome-wide CRISPR/Cas9-based screen and the strategy used to magnetically enrich for K562 library cells that lost expression of KIR3DS1 ligands. **(C)** Flow cytometry histograms of K562 library cells before (*top panel*) and after (*bottom panel*) magnetic enrichment are shown. **(D)** Dot plot depicting enrichment of sgRNA-targeted genes calculated from sequencing analysis of the enriched cell population. CRISPR score calculation is described in Material and Methods** **. Each dot indicates a particular gene, and each alternating section of black and gray dots indicates individual chromosomes (chromosomes 1 – 22 and X+Y). Large red dots indicate top nine enriched genes (hits), all of which were involved in heparan sulfate biosynthesis. **(E)** Diagram of heparan sulfate biosynthesis (structure and enzymes); the top nine hits are indicated in red bolded text.

We stained several cell lines with a soluble fusion chimera consisting of the extracellular domain of KIR3DS1 attached to the Fc region of human IgG1 (KIR3DS1-Fc). We chose KIR3DS1 because it does not bind to HLA class I or class II genes [except HLA-F, which has restricted tissue expression (20, 21)]. Surprisingly, KIR3DS1-Fc bound to all human cell lines arising from many cell lineages including those of T-cell (Jurkat), B-cell (721.221, RAJI, and EBV-transformed primary B-cell line), monocytic (THP-1), and erythro-myeloid (K562) origin (**Figure 1A**). Of interest, K562 cells are HLA class I deficient (including HLA-F deficient), which cued us to the existence of non-HLA ligands for KIR3DS1.

### Genome-Wide CRISPR/Cas9 Knock-Out Screen Reveals That Heparan Sulfate Biosynthesis Enzymes Are Critical for Binding of KIR3DS1

Given the widespread expression of KIR3DS1 ligands across various human cell lines, we decided to perform a pooled, genome-wide CRISPR/Cas9-based screen [previously described in (22)] in K562 cells, which are HLA-deficient and exhibited the highest levels of KIR3DS1-Fc binding (**Figure 1B**). Briefly, K562 cells stably expressing Cas9 were transduced with a lentiviral single-guide RNA (sgRNA) library that targeted 18,166 human protein-coding genes, and were then cultured under selection to allow for gene inactivation and turnover of gene products to occur. K562 library cells were then stained with KIR3DS1-Fc, followed by a secondary stain with a PE-conjugated anti-human IgG (Fc-specific) antibody, and a tertiary stain with a magnetic-bead–conjugated anti-PE antibody to perform magnetic enrichment. The magnetically enriched cells were cultured, expanded, and then analyzed by flow cytometry, which showed that whereas almost all cells in the initial population (i.e. pre-enrichment) bound to KIR3DS1-Fc, cells cultured post-enrichment had ~50% of cells staining very dimly for KIR3DS1-Fc binding (**Figure 1C**).

Using high-throughput sequencing, sgRNA barcodes in the cultured post-enrichment cell population were quantified to assess enrichment of specific sgRNA sequences. Remarkably, sequencing showed that the top nine enriched target genes encoded enzymes involved in heparan sulfate biosynthesis (**Figure 1D** and **Table 1**). These enzymes were directly involved in either heparan sulfate polysaccharide backbone polymerization (i.e. XYLT2, B4GALT7, B3GAT3, EXTL3, EXT1, EXT2), or in the synthesis of precursor molecules needed for heparan sulfate biosynthesis (i.e. UXS1, UGDH, and PAPSS1) (**Figure 1E**). These results indicated that heparan sulfate proteoglycans were a target ligand of KIR3DS1-Fc on K562 cells.

**Table 1:** Genome-wide CRISPR/Cas9-based screen top hits.

Gene Symbol	Description	CRISPR Score
**EXT1**	exostosin glycosyltransferase 1	7.047
**UXS1**	UDP-xylose synthase 1	4.949
**EXTL3**	exostosin-like glycosyltransferase 3	4.394
**UGDH**	UDP-glucose 6-dehydrogenase	4.032
**PAPSS1**	3’-phosphoadenosine 5’-phosphosulfate synthase	1.150
**EXT2**	exostosin glycosyltransferase 2	0.712
**B3GAT3**	beta-1,3-glucuronyltransferase 3	0.642
**XYLT2**	xylosyltransferase 2	0.473
**B4GALT7**	xylosylprotein beta 1,4-galactosyltransferase 7	–0.495

Top nine enriched sgRNA gene targets (symbol and description) are presented with their calculated CRISPR scores.

### Surface Plasmon Resonance Shows That KIR3DS1 and Other KIRs That Contain a D0 Domain Bind to Heparan Sulfate

**Figure 2:**
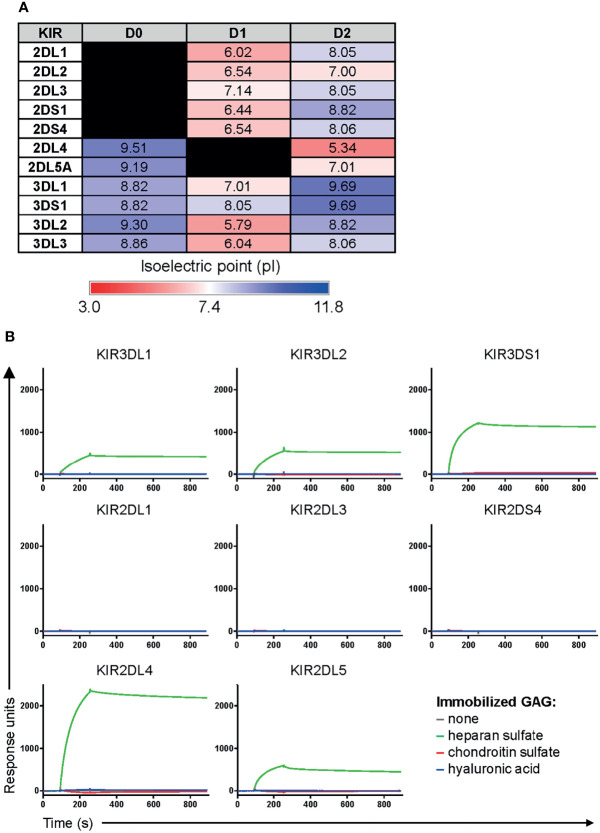
KIRs that contain a D0 domain bind heparan sulfate. **(A)** The isoelectric point (pI) of individual domains and regions of the indicated KIRs was calculated using the ExPASy Compute pI/Mw tool (24). This was used as a rough estimate of the net charge status of each domain/region. **(B)** Surface plasmon resonance sensograms of Fc constructs of the indicated KIRs (at 25 μg/mL) flowed over immobilized heparan sulfate, chondroitin sulfate, or hyaluronic acid for 3 min, and then allowed to dissociate by flowing over buffer for 10 min.

Given that protein interactions with heparan sulfate are dominated by electrostatic forces between positively-charged basic residues on proteins and negatively-charged sulfate moieties on heparan sulfate (6, 23), we computationally determined the isoelectric point (pI) of various domains and regions of KIR3DS1 and other KIRs to roughly assess their potential for interacting with heparan sulfate. Our analyses revealed that D0 domains, which only some KIRs (including KIR3DS1) contain, have a pI above physiological pH (**Figure 2A**), indicating that the D0 domain of these KIRs has a positive net charge and likely mediates binding to heparan sulfate.

To confirm this, we performed surface plasmon resonance on several KIRs to comparatively assess their affinities to heparan sulfate. We included two additional GAGs, hyaluronic acid and chondroitin sulfate, as controls. Remarkably, we found that all Fc constructs of KIRs containing a D0 domain (KIR3DS1, KIR3DL1, KIR3DL2, KIR2DL4, and KIR2DL5) bound to heparan sulfate but not to chondroitin sulfate or hyaluronic acid, whereas there was no GAG binding observed for KIRs not containing a D0 domain (KIR2DL1, KIR2DL3, and KIR2DS4) (**Figure 2B**). Interestingly, kinetic analyses revealed that these interactions were somewhat higher affinity than previously published affinities of KIR3DS1, KIR3DL1, and KIR3DL2 towards HLA-F open conformers (20) (**Table 2**). This was in concordance with the results from the CRISPR/Cas9 screen and our prediction of KIRs containing a D0 domain having a unique ability to bind to heparan sulfate *via* electrostatic interactions.

**Table 2:** Kinetic analyses of KIR-Fc binding to heparan sulfate.

	*k_a_* (M^–1^s^–1^) (× 10^4^)	*k_d_* (s^–1^) (× 10^–4^)	*K_D_* (nM)
KIR3DS1	7.22 ± 0.004	2.2 ± 0.2	3.1 ± 0.3
KIR3DL1	2.29 ± 0.0002	1.04 ± 0.05	4.5 ± 0.2
KIR3DL2	3.22 ± 0.0007	2.0 ± 0.2	6.2 ± 0.6
KIR2DL4	3.54 ± 0.001	6.29 ± 0.06	17.8 ± 0.2
KIR2DL5	3.82 ± 0.01	16.1 ± 0.09	42.3 ± 0.4

Kinetic analyses were performed on KIR-Fc constructs that demonstrated binding to heparan sulfate by surface plasmon resonance. The following monomeric interaction kinetic values based on a bivalent analyte model are presented (fitted value ± standard error): k_a_, association rate constant (‘on’ rate); k_d_, dissociation rate constant (‘off’ rate); and K_D_, equilibrium dissociation constant.

### Elimination of Heparan Sulfate Proteoglycans on Cell Lines Abrogates KIR Binding Unless Canonical Protein Ligands Are Present

**Figure 3:**
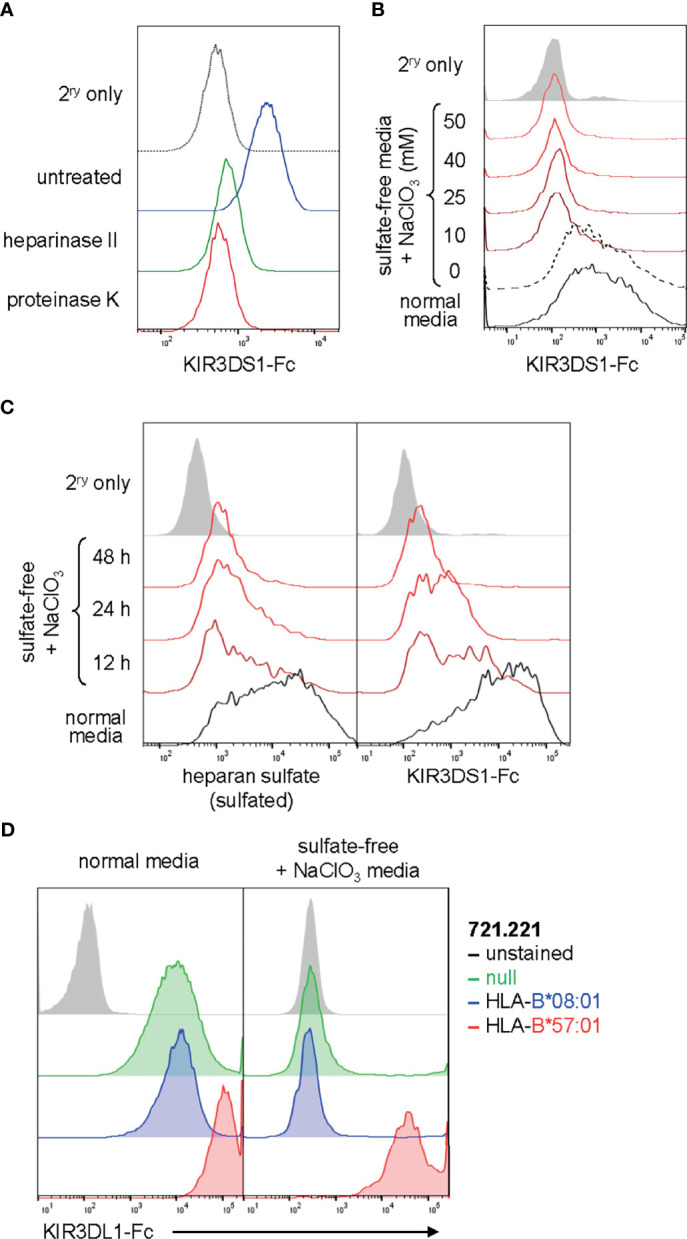
KIR binding to heparan sulfate on cells can be abrogated by heparan sulfate degradation or sulfation inhibition and is independent of binding to known HLA ligands. **(A)** Flow histogram of 721.221 cells treated with heparinase II, proteinase K, or no enzyme and stained with KIR3DS1-Fc (25 μg/mL). **(B)** EBV-BCL cells were cultured for 48 h in sulfate-free media containing the indicated concentrations (in mM) of NaClO_3_, and then stained with KIR3DS1-Fc (25 μg/mL). **(C)** Human donor-derived EBV-BCL cells were cultured for the indicated amounts of time with sulfate-free media containing 50 mM NaClO_3_ or regular media and separately stained with anti-heparan sulfate antibody (clone: 10E4) and KIR3DS1-Fc (25 μg/mL). **(D)** 721.221 cells that were untransduced or transduced with HLA-B*08:01 or HLA-B*57:01 were cultured in regular media or in sulfate-free/NaClO_3_-containing media and stained with KIR3DL1-Fc (25 μg/mL).

To further validate our findings, we investigated whether KIR3DS1 binding to cell lines can be abolished by eliminating heparan sulfate proteoglycans on the cell surface. To accomplish this, we treated 721.221 cells, another HLA-deficient cell line that showed KIR3DS1-Fc binding (**Figure 1A**), enzymatically with heparinase II or proteinase K. Heparinase II degrades the heparan sulfate sugar backbone while proteinase K cleaves cell-surface proteins (including the proteoglycans to which heparan sulfate is attached) without significantly affecting cell integrity. Indeed, enzymatic treatment with both heparinase II and proteinase K resulted in a dramatic decrease in KIR3DS1-Fc binding to 721.221 cells compared to untreated controls, consistent with heparan sulfate proteoglycans being a cellular ligand for KIR3DS1-Fc (**Figure 3A**).

Given that *PAPSS1* scored highly in the CRISPR/Cas9-based screen, we were also interested in experimentally determining whether binding of KIR3DS1 to surface-expressed heparan sulfate proteoglycans was dependent on the presence of sulfate moieties on heparan sulfate. *PAPSS1* encodes for 3’-phosphoadenosine-5’-phosphosulfate (PAPS) synthase, an enzyme required to generate PAPS, which is the universal sulfate donor for all cellular sulfotransferase reactions. PAPS synthase can be chemically inhibited by chlorate $\left(\text{Cl}{\text{O}}_{3}^{-}\right)$ , an inhibitor that competitively blocks binding of sulfate ions. To eliminate sulfate moieties from heparan sulfate, we cultured EBV-BCLs, which also exhibited high levels of KIR3DS1-Fc binding, in sulfate-free media containing different concentrations of $\text{Cl}{\text{O}}_{3}^{-}$ (ranging from 10 to 50 mM) and found that KIR3DS1-Fc binding was lost even at low concentrations of $\text{Cl}{\text{O}}_{3}^{-}$ (**Figure 3B**). KIR3DS1 binding and heparan sulfation content were then assessed at various time points after culturing cells in sulfate-free media containing $\text{Cl}{\text{O}}_{3}^{-}$ . Heparan sulfation content was measured using an anti-heparan sulfate antibody (clone: 10E4) that recognizes sulfated GlcN residues (25). Continuous culture in sulfate-free media containing $\text{Cl}{\text{O}}_{3}^{-}$ resulted in loss of cell-surface heparan sulfation and a concomitant decrease in KIR3DS1-Fc binding (**Figure 3C**). This demonstrated that binding of KIR3DS1 to heparan sulfate is dependent on the presence of sulfate moieties, which has been shown to be the case for other heparan sulfate-binding proteins (16). One previous report demonstrated that $\text{Cl}{\text{O}}_{3}^{-}$ concentrations of 5–20 mM resulted in selective loss of heparan sulfate 6-*O*-sulfate moieties (26), which may implicate these moieties specifically in binding of KIR3DS1 to heparan sulfate.

Finally, we wanted to test whether KIR binding to heparan sulfate proteoglycans has an impact on binding to known canonical ligands (e.g. HLA). To do so, we explored the effects of heparan sulfate elimination on the interaction of KIR3DL1 with its ligand HLA-B*57:01. We performed KIR3DL1-Fc staining of 721.221 cells that were untransduced (null) or stably transduced with HLA-B*08:01 (a non-ligand) or HLA-B*57:01 (a known HLA ligand) and cultured in either normal media or sulfate-free medium containing $\text{Cl}{\text{O}}_{3}^{-}$ . Remarkably, we found that while elimination of heparan sulfate proteoglycans abolished binding of KIR3DL1-Fc to untransduced and HLA-B*08:01-expressing 721.221 cells, binding to 721.221 cells expressing HLA-B*57:01 was maintained at a high level (**Figure 3D**). This supports the notion that KIR binding to HLA is not significantly affected by the absence of heparan sulfate proteoglycans.

## Discussion

Using a genome-wide CRISPR/Cas9 knock-out screen, we identified heparan sulfate proteoglycans as ligands for the activating NK-cell receptor KIR3DS1 on K562 cells. KIR3DS1 binding to heparan sulfate was confirmed *via* surface plasmon resonance and on different cell lines, and predicted to be the dependent on its D0 domain mediating favorable electrostatic interactions. These analyses were extended to other KIRs, resulting in the finding that all tested KIRs containing a D0 domain bound heparan sulfate.

Several different approaches to identify novel or additional ligands for NK-cell receptors have been employed in the past. Our group previously discovered *via* an HLA class I screen that KIR3DS1 binds HLA-F open conformers, a finding that was confirmed by others and was validated biochemically and functionally (20, 27). In the current study, ligand-screening was performed using K562 cells, a human erythromyeloid leukemia cell line devoid of HLA class I gene products. This explains why HLA-F was not identified as a target using the approach performed here, despite HLA-F sgRNAs being present in the sgRNA library. However, the genome-wide CRISPR/Cas9-based screen allowed us to unbiasedly identify KIR3DS1 ligands of any nature as long as they were expressed at the cell surface in the cell line of choice and not encoded by redundant genes.

The binding of KIRs to heparan sulfate proteoglycans described here has important implications. Indeed, whereas KIR2DL4 was previously shown to interact with heparan sulfate *via* its D0 domain (19), other NK-cell receptors with D0 domains, namely, KIR2DL5 and all KIR3D family members, were not previously examined for heparan sulfate-binding properties to the best of our knowledge. Interestingly, we found that KIR2DL5, which was recently found to bind to the poliovirus receptor (PVR) (28), also bound to heparan sulfate, which explains why KIR2DL5-Fc fusion proteins exhibit “*dull staining of, essentially, every human cell line [… ] independent of the cells HLA allotypes*” (29), as described by other investigators. Both KIR3DL1 and KIR3DS1 also bound to heparan sulfate, but KIR3DS1, a highly homologous activating allotype of KIR3DL1 with >97% extracellular domain sequence identity, bound to heparan sulfate to a greater extent on surface plasmon resonance, which may be explained by the critical L166R mutation that abrogates KIR3DS1 binding to HLA-Bw4^I80^ proteins (30), yet increases the net positive charge of its D1 domain. It is also possible that KIR3DL3, which was recently found to bind HHLA2 (31), may have similar properties, but this needs to be formally tested in future studies. In general, understanding and eliminating the heparan sulfate-binding properties of KIRs and other receptors is a very useful adjunctive strategy for investigators seeking to perform cell-based ligand screens with clear-cut detection of other receptor-ligand interactions.

Interactions of KIRs and other NK-cell receptors in *trans* with heparan sulfate expressed on target cells represent a potentially unexplored mechanism of target cell recognition. Indeed, specific cell types (and cellular contexts) exhibit precise compositions of cell-surface heparan sulfate proteoglycans, also referred to as the “heparanosome” signatures. These contain a large variety of interaction motifs for heparan sulfate-binding proteins. Cancer cells of various tissue origins exhibit significantly deregulated expression of heparan sulfate biosynthesis genes, which has been shown to play a role in tumorigenesis affecting various biological processes that are hallmarks of cancer (such as tissue invasion and altered growth factor signaling) (32). Interestingly, it has been observed that cancers that modulate heparan sulfate by methylation-associated silencing of sulfotransferase genes exhibit progression and poor prognosis (33, 34). These heparanosome changes that occur in tumor cells may result in enhanced engagement with immune receptors, supported by the fact that we see widespread high-level binding of KIR3DS1 to all tested tumor cell lines. Thus, it is conceivable that alterations in heparan sulfate proteoglycans that occur during tumorigenesis can lead to increased NK-cell recognition (or evasion), and our finding that multiple NK-cell receptors interact with heparan sulfate proteoglycans supports further investigating this possibility.

*Cis* interactions between heparan sulfate and NK-cell receptors like KIR2DL4 have been shown to cause clustering and block *trans* interactions, allowing for an additional level of tunable control over NK-cell–receptor signaling (19). It is conceivable that upon cellular activation, NK cells alter the composition of cell-surface heparan sulfate to alter receptor signaling and functionality. This change in cell-surface composition has been described for CD57, an epitope comprising a surface-expressed glucuronic acid 3-*O*-sulfate moiety produced by the enzyme B3GAT1 (35). Expression of CD57 has been seen to occur in NK cells with a “mature” phenotype characterized by poor proliferative capacity, poor cytokine responsiveness, and increased sensitivity to CD16-mediated activation (36). While the exact consequences of changes in the heparanosome on NK cells still remain to be determined, our data provide first insights into a global binding potential of KIR interactions with heparan sulfate that could have an important influence on the signaling capacity of receptors and overall NK-cell functionality.

In addition, there is also consideration to be given to binding of NK-cell receptors to secreted heparan sulfate that forms part of extracellular matrix (ECM). It is possible that this form of heparan sulfate may serve as a “decoy” that interrupts intercellular interactions. One study demonstrated that both murine and human NK cells express heparanase upon activation, which was necessary for tumor invasion and prevention of metastases (37). It is tempting to speculate that in addition to facilitating tumor infiltration, NK cells degrade ECM heparan sulfate to favor NK-cell receptor interactions with target cell ligands, although this has yet to be determined.

Overall, we demonstrate the feasibility of performing a genome-wide CRISPR/Cas9 knock-out screen to identify previously unknown ligands of NK-cell receptors, showing the existence of an interaction between several KIRs and heparan sulfate proteoglycans. This interaction may play a wide-ranging role in regulating NK-cell function and immunosurveillance, and studies that further explore the extent and specificity of these interactions may allow for further understanding and manipulating NK-cell biology.

## Materials and Methods

### Cell Lines

K562, Jurkat, THP-1, RAJI, and 721.221 cell lines (including HLA transductants) were grown in RPMI-1640 supplemented with 10% fetal bovine serum (Sigma-Aldrich), 2 mM L-glutamine (Gibco), 100 U/mL penicillin (Gibco), and 100 U/mL streptomycin (Gibco) at 37°C/5% CO_2_. EBV-transformed B-cell lines (EBV-BCLs) were generated from peripheral blood mononuclear cells from donors bearing specific HLA genotypes; EBV-BCLs were also grown in the same media and conditions. Human samples were used in this study in accordance to protocols approved by Partners Human Research Committee and Institutional Review Board of Massachusetts General Hospital.

### KIR-Fc Constructs and Antibodies

All KIR-Fc constructs used in this study were produced in mammalian cell lines and purchased from R&D Systems; they included the following: KIR3DS1-Fc, KIR3DL1-Fc, KIR3DL2-Fc, KIR2DL1-Fc, KIR2DL3-Fc, KIR2DL4-Fc, KIR2DS4-Fc, and KIR2DL5-Fc. Cells were stained with KIR-Fc constructs by washing extensively with PBS, incubating for 30–45 min with 25 μg/mL KIR-Fc diluted in PBS at 4°C while shaking, followed by a secondary staining with goat anti-human lgG Fc-PE (Life Technologies) for 30 min at 4°C while shaking. After staining, cells were washed and fixed using 4% paraformaldehyde/PBS (Affymetrix) and flow cytometric analysis was performed on an BD LSR II or LSR Fortessa. Measurement of heparan sulfate expression by flow cytometry was done using anti-HS-FITC (clone: 10E4, US Biological; used at 1:10 dilution).

### Magnetic Enrichment-Based Genome-Wide CRISPR/Cas9-Based Screen

CRISPR/Cas9-based library of pooled mutant cells was generated similarly to previously described work published in (22). *sgRNA Library design.* The optimized sgRNA library was designed by first computationally filtering out potential off-target matches (except for sgRNAs that targeted multiple homologs), and also to have high cleavage activity (based on rules experimentally determined in another set of experiments). In total, a library containing 178,896 sgRNAs targeting 18,166 protein-coding genes in the human consensus CDS (CCDS) and 1,004 non-targeting control sgRNAs was constructed. *Cell library generation.* K562 cells were lentivirally transduced with Cas9/eGFP vector. 2.40 × 10^8^ Cas9-transduced cells were transduced with the viral pool containing the sgRNA library to achieve an average 1000-fold coverage of the library after selection with puromycin for 7 d. *Screening procedure*. 5 × 10^7^ pooled mutant K562 cells were washed in PBS and stained in 1 mL of 25 μg/mL KIR3DS1-Fc (R&D) for 15 min at 4°C. Cells were then washed with PBS and stained with 1 mL of anti-hIgG(Fc)-PE (Life Technologies) diluted 1:50 in PBS. Fluorescently labeled cells were washed with PBS and stained with anti-PE Microbeads (Miltenyi) following manufacturer’s instructions but using twice the recommended volumes. Magnetically labelled cells were then depleted in an LD column (Miltenyi) following manufacturer’s instructions, and flow-through cells were collected and cultured for 7 d. *Screen deconvolution.* sgRNA inserts were PCR amplified from 5 million genome equivalents of DNA from flow-through cells. The resultant PCR products were purified and sequenced on a HiSeq 2500 (Illumina); primer sequences are as follows:

sgRNA quantification primers:

F: AATGATACGGCGACCACCGAGATCTAGAATACTGCCATTTGTCTCAAG
R: CAAGCAGAAGACGGCATACGAGATCnnnnnnTTTCTTGGGTAGTTTGCAGTTTT (nnnnn denotes the sample barcode)

Illumina sequencing primer:

CGGTGCCACTTTTTCAAGTTGATAACGGACTAGCCTTATTTTAACTTGCTATTTCTAGCTCTAAAAC

Illumina indexing primer:

TTTCAAGTTACGGTAAGCATATGATAGTCCATTTTAAAACATAATTTTAAAACTGCAAACTACCCAAGAAA

*Data analysis.* Sequencing reads were aligned to the sgRNA library and the abundance of each sgRNA was calculated. Gene-based CRISPR scores (CS) were defined as the average log2 fold change of all sgRNAs targeting a given gene and calculated for the entire screen. To identify enriched genes, the CS distribution was mean-normalized to zero.

### Sulfation Inhibition and Enzymatic Treatment of Cell Lines

For sulfation inhibition, cells were grown for 48 h (or other time period if specified) in sulfate-free/NaClO_3_-containing media, which consisted of custom-made Advanced RPMI-1640 (Life Technologies) which had 407 μM magnesium sulfate replaced with 407 μM magnesium chloride, 3.03 μM zinc sulfate replaced with 3.03 μM zinc chloride, 5 nM copper (II) sulfate replaced with 5 nM copper (II) chloride, and sodium chloride reduced from 103 mM to 53 mM, and was supplemented with 10% dialyzed FBS (Gibco), 2 mM L-glutamine (Gibco), 100 μg/mL primocin (*In vivo*gen), and 50 mM sodium chlorate (NaClO_3_; Sigma-Aldrich) or other NaClO_3_ concentration if specified, which was balanced with sodium chloride (Sigma-Aldrich).

For surface enzymatic treatment of cells, 2.5 × 10^5^ cells were washed three times with PBS, and then incubated in PBS containing 2 U/mL heparinase II (New England BioLabs), 1 U/mL proteinase K (New England BioLabs), or no enzyme for 1 h at 37°C/5% CO_2_. Cells were then washed in ice-cold PBS and stained as indicated.

### Computational Analysis of pI

Isoelectric point analysis was determined by inputing Ig-domain sequences (from Cys to Cys) into ExPASy Compute pI/Mw tool, which calculates pI using pK_a_ values of amino acids (24). The KIR sequences analyzed were obtained from Immuno Polymorphism Database (IPD) (38, 39) and were the following: KIR2DL1*001, KIR2DL2*001, KIR2DL3*001, KIR2DL4*001, KIR2DL5A*001, KIR2DS1*002, KIR2DS4*001, KIR3DL1*001, KIR3DS1*013, KIR3DL2*001, and KIR3DL3*001.

### Surface Plasmon Resonance (SPR)

SPR measurements were conducted in phosphate-buffered saline (PBS; Corning) containing 0.005% v/v surfactant P20 (GE Healthcare) using a Biacore 3000 system (Biacore AB). To assess binding of various KIR-Fc constructs (R&D) to various GAGs, biotinylated hyaluronate (molecular weight = 29 kDa), biotinylated heparin (molecular weight = 18 kDa) and biotinylated chondroitin sulfate (molecular weight = 50 kDa) (all from Creative PEGWorks), were immobilized onto individual flow cells of an SA (streptavidin) sensor chip (GE Healthcare) until saturation. A blank flow cell with no immobilized ligand was used as a reference flow cell. Injections of 60 μL of KIR-Fc constructs diluted in PBS to 25 μg/mL were performed at a flow rate of 20 μL/min, with a subsequent 10 min run of buffer to allow sufficient dissociation. Although not presented here, regeneration after each injection was achieved with two pulses of 100 μL of 0.2 M sodium hydroxide (NaOH) (GE Healthcare) at a flow rate of 100 μL/min. Raw sensograms were corrected by double referencing (subtracting from the reference flow cell response and from PBS injection response). All experiments were done at standard temperature (25°C).

### Data Acquisition and Analysis

Flow cytometry data was acquired on BD LSR II or LSR Fortessa and analyzed using FlowJo software version 10.1 (FlowJo) and analyses were performed using GraphPad Prism 6 (GraphPad Software). Kinetic analyses of SPR data were performed using TraceDrawer 1.9.2 software (Ridgeview Instruments AB) using bivalent analysis model (due to use of dimeric KIR-Fc) to calculate monomeric affinities.

## Data Availability Statement

The datasets presented in this study are deposited in the Mendeley Data repository, accessible by https://data.mendeley.com/datasets/3k2rtfv9bx/1.

## Author Contributions

KK, WG-B, T-EK, and HD performed experiments and analyzed data. TW provided CRISPR screening tools and analyzed deep sequencing data. AH, SJ, and MA provided significant input regarding the design and execution of this work. KK and WG-B wrote the paper. All authors contributed to the article and approved the submitted version.

## Funding

This work was supported by U.S. National Institutes of Health (R01-AI067031-08 and P01-AI104715; F31AI116366 to WG-B), the National Institute of General Medical Sciences (T32GM007753), Ragon Institute of MGH, MIT and Harvard, Leibniz Institute for Experimental Virology (Program Area Antiviral Targets and Strategies), and German Center for Infection Research (DZIF) (TTU 01.709;8009701709 for AH).

## Author Disclaimer

The content is solely the responsibility of the authors and does not necessarily represent the official views or policies of the above-mentioned organizations, nor does mention of trade names, commercial products, or organizations imply endorsement by the U.S. or German Government.

## Conflict of Interest

Author HD is employed by Adimab, LLC.

The remaining authors declare that the research was conducted in the absence of any commercial or financial relationships that could be construed as a potential conflict of interest.

## Publisher’s Note

All claims expressed in this article are solely those of the authors and do not necessarily represent those of their affiliated organizations, or those of the publisher, the editors and the reviewers. Any product that may be evaluated in this article, or claim that may be made by its manufacturer, is not guaranteed or endorsed by the publisher.
